# Intraosseous Non‐Hodgkin Lymphoma Mimicking a Periapical Lesion

**DOI:** 10.1111/scd.70162

**Published:** 2026-03-27

**Authors:** Laryssa Thainá Mello Queiroz Cunha, Joab Cabral Ramos, Isabel Schausltz Pereira Faustino, Silvia Maria Paparotto Lopes, Alan Roger Santos‐Silva, Pablo Agustin Vargas, Márcio Ajudarte Lopes

**Affiliations:** ^1^ Department of Oral Diagnosis, Stomatology and Oral Pathology Areas, Piracicaba Dental School State University of Campinas (UNICAMP), Piracicaba São Paulo Brazil; ^2^ Department of Stomatology, Public Health and Legal Dentistry University of Sao Paulo (USP), Ribeirão Preto São Paulo Brazil; ^3^ Private Dental Practice, Piracicaba São Paulo Brazil

**Keywords:** endodontic diagnosis, lymphoma, non‐endodontic pathology, periapical lesion

## Abstract

**Introduction:**

Periapical radiolucent lesions are commonly of endodontic origin; however, some persistent cases may indicate non‐endodontic pathologies, including malignancies. Diffuse large B‐cell lymphoma (DLBCL) is the most prevalent subtype of lymphoma affecting the oral and maxillofacial region, yet its primary occurrence in the jaws is rare. This case report highlights the diagnostic challenges in differentiating endodontic lesions from periapical lymphomas and includes a literature review of documented cases.

**Case Report:**

A 44‐year‐old male presented with a persistent periapical lesion in the anterior maxilla, unresponsive to endodontic and periodontal treatments. Clinical examination revealed facial asymmetry, gingival edema, palatal swelling, and dental mobility. Imaging showed an ill‐defined hypodense lesion extending beyond the periapical region. Fine needle aspiration and incisional biopsy confirmed DLBCL through histopathology and immunohistochemistry. The patient underwent six cycles of R‐CHOP chemotherapy.

**Results:**

After treatment, intraoral examination revealed mucosal integrity, absence of swelling, and stabilization of dental mobility. Cone‐beam computed tomography demonstrated bone regeneration, and PET/CT imaging confirmed complete remission.

**Conclusion:**

This case underscores the importance of considering malignancies in the differential diagnosis of persistent periapical lesions. Accurate clinicoradiographic assessment, histopathological confirmation, and interdisciplinary collaboration are essential for timely diagnosis and improved patient outcomes.

## 1 Introduction

Most periapical radiolucent lesions result from dental pulp necrosis and are classified as periapical lesions of endodontic origin [1]. In most cases, these lesions heal, and bone regeneration occurs after endodontic treatment [2]. However, the periapical region can also be affected by non‐endodontic lesions, which include a wide variety of pathologies such as cysts, benign and malignant tumors, fibro‐osseous lesions, and bacterial or fungal infections [1, 3]. The literature highlights that the clinical and radiographic similarity between endodontic and non‐endodontic lesions often complicates diagnostic differentiation, potentially leading to misdiagnosis and inappropriate treatment [4].

Among the lesions that may mimic endodontic pathologies, lymphomas should be considered in the differential diagnosis, particularly diffuse large B‐cell non‐Hodgkin lymphoma (DLBCL), the most common subtype of lymphoma affecting the oral cavity [3, 5]. Although lymphomas can involve the oral cavity and jawbones, their primary occurrence in these structures is rare, making definitive diagnosis challenging [5]. Patients with periapical lymphoma may present with signs and symptoms such as swelling, persistent pain, tooth mobility, and bone resorption, features often mistakenly attributed to endodontic diseases [6]. Additionally, these lesions may exhibit nonspecific radiographic characteristics, typically appearing as osteolytic areas with ill‐defined borders, which may delay diagnosis and negatively impact the patient's prognosis [7].

Given this scenario, the present case report describes a patient with DLBCL initially manifesting as a persistent periapical lesion in the anterior maxillary region. Moreover, we provide a comprehensive review of lymphomas occurring in the periapical region reported in English‐language literature. The literature review was conducted through a systematic search of the PubMed database, using combinations of terms related to diffuse large B‐cell non‐Hodgkin lymphoma (DLBCL), periapical lymphoma, malignant periapical lesions, and pathologies mimicking endodontic conditions. The search strategy was adapted according to the database indexing, with no restriction on publication date. Additionally, a manual search of the references of included studies was performed to identify further relevant articles. Duplicates were removed during the screening phase to ensure the inclusion of unique and pertinent studies for analysis. Two reviewers independently performed the initial screening of studies based on titles and abstracts, followed by full‐text reading. Any disagreements were resolved by a third reviewer. The Rayyan QCRI software was used for reference management, duplicate removal, and documentation of exclusion reasons. Data extraction was conducted by the first reviewer and subsequently verified by another, including the following information: author/year, age and gender of the population, diagnosis, performance of endodontic treatment, and time to diagnosis.

This study aims to emphasize the importance of recognizing the key characteristics of malignant periapical lesions, particularly lymphoma, by general practitioners and endodontists, as well as the necessity of establishing a differential diagnosis with lesions of endodontic origin.

## 2 Case Report

A 44‐year‐old male was referred to the Oral Medicine service for evaluation of an intraosseous lesion in the maxillary region. According to the patient's history, the initial evaluation and treatment were performed by an external private dentist approximately 60 days earlier, after the onset of intense pain in the upper left lateral incisor. At that time, a periapical radiograph was obtained, showing a diffuse radiolucent area in the periapical region of tooth #7. Clinical assessment included pulp vitality testing, which was negative, supporting the indication for endodontic treatment. Root canal therapy was then performed by the same provider. Following the endodontic procedure, the patient developed gingival edema and palatal swelling, and the same dentist carried out periodontal scaling and root planning associated with photobiomodulation therapy. Only after persistence and progression of the signs and symptoms was the patient referred to the Oral Medicine service for further investigation and management. His medical history included systemic hypertension, treated with BenicarAnlo. The patient denied alcohol, tobacco, or any other significant habits.

**FIGURE 1:**
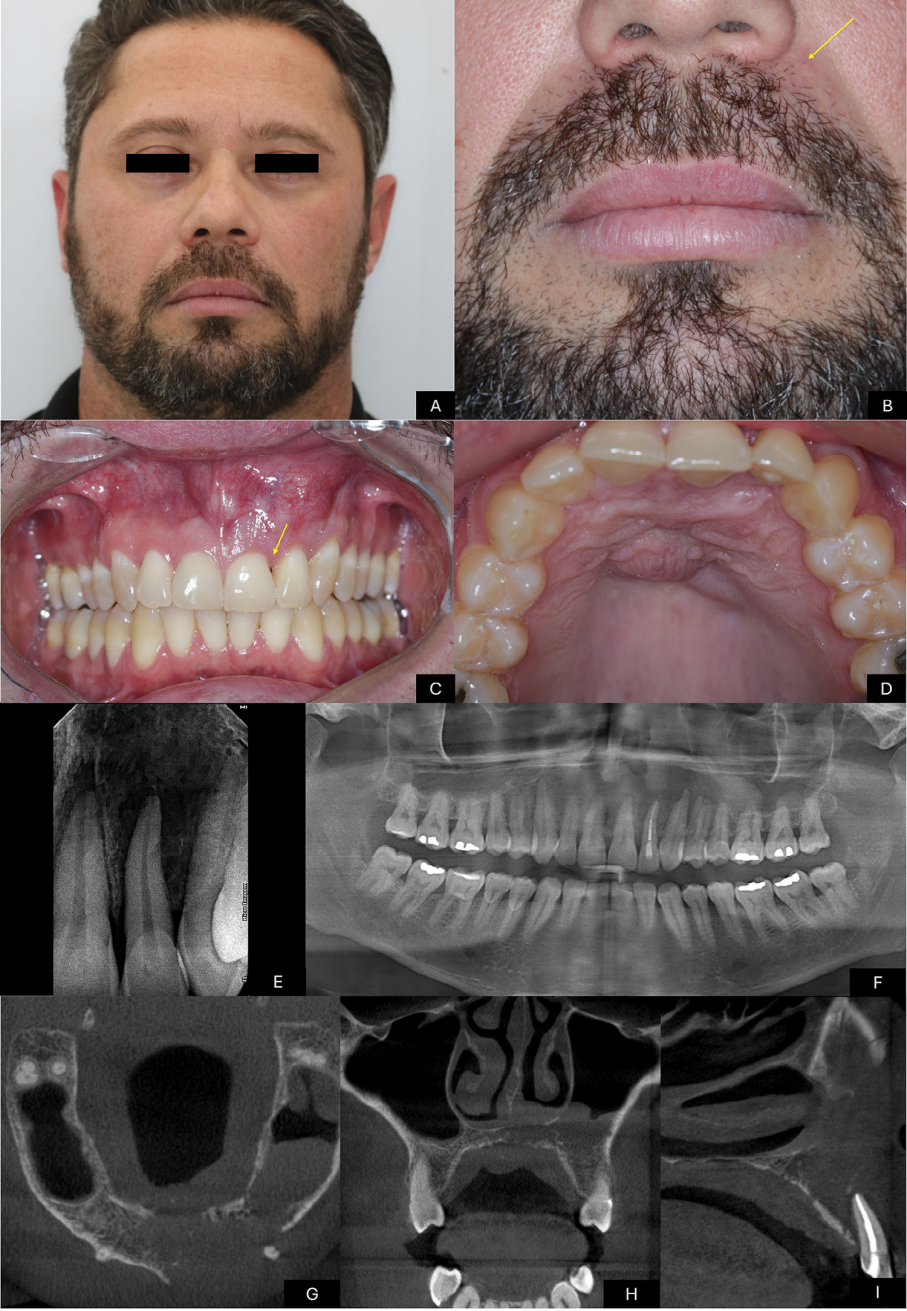
Initial clinical and radiographic aspects. (A‐B) Initial clinical examination revealing mild facial asymmetry due to a localized swelling in the upper left lip region (yellow arrow). (C) Intraoral swelling in the anterior region of the left maxilla, resulting in a reduced maxillary vestibular fornix, with a purplish hue and a telangiectatic surface. A decrease in the interdental papilla is observed in the interproximal region between teeth lateral and central incisors (yellow arrow). (D) Swelling along the midline of the hard palate. (E) Periapical radiograph demonstrating a diffuse radiolucent area at the periapex of tooth #7. (F) Panoramic radiograph showing a poorly defined radiolucent image in the periapical region of teeth. Tooth lateral incisor presents previous endodontic treatment. (G) Axial slice of computed tomography (CT) revealing an expansile hypodense lesion in the anterior maxillary region, associated with cortical bone disruption. (H) Coronal CT reconstruction demonstrating hypodensity in the left maxillary sinus and the inferior portion of the nasal cavity, associated with bone destruction. (I) Sagittal CT reconstruction showing satisfactory endodontic treatment of tooth lateral incisor, associated with bone destruction, cortical disruption, and root resorption.

Upon physical examination, the patient appeared to be in good general health, with stable vital signs. However, facial asymmetry was observed in the left maxillary region. Intraoral examination revealed fibroelastic swelling in the anterior region of the left maxilla, resulting in a reduced maxillary vestibular fornix, with a purplish hue and a telangiectatic surface. In the interproximal region between central and lateral incisors, a reduction in the interdental papilla was observed, creating a “black space.” Additionally, a volume increase of approximately 1.5 cm was noted along the midline of the hard palate, with a smooth surface, a mucosa‐like color, and no tenderness upon palpation. The central and lateral incisors, canine and first premolar exhibited grade II mobility, with no fistulas or purulent discharge. Oral hygiene was adequate, and no odontogenic infectious foci were identified. A panoramic radiograph showed an imprecise radiolucent area on the periapical region of these teeth. Computed tomography (CT) scan revealed a hypodense lesion; with ill‐defined borders with involvement of the left maxillary sinus and the inferior portion of the nasal cavity, with important bone destruction (Figure 1) (Supplementary Material).

**FIGURE 2:**
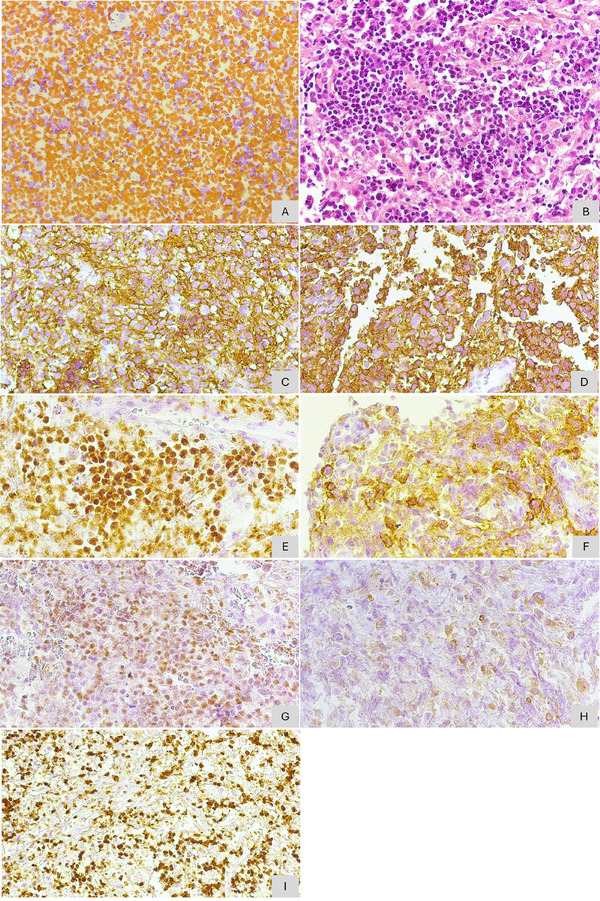
Microscopic analysis demonstrating connective tissue infiltrated by neoplastic cells. (A) Fine‐needle aspiration (FNA) smear revealing large, round cells with frequent mitotic figures. (B) Hematoxylin‐eosin staining showing atypical lymphoid cells with hyperchromatic nuclei and atypical mitotic activity. Immunohistochemical staining demonstrating positivity for (C) LCA, (D) CD20, (E) PAX5, (F) CD10, (G) BCL‐6 (>30%), (H) BCL‐2 (<30%), and (I) a high proliferative index with Ki‐67 expression at 80%.

Based on the clinical history and imaging findings, the main differential diagnoses included malignancy, particularly lymphoma or sarcoma. Fine needle aspiration (FNA) and incisional biopsy were performed under local anesthesia. For the incisional biopsy, a vestibular incision was made in the anterior maxilla, in the region of the left central and lateral incisors. A fragment of soft tissue was removed and submitted for histopathological analysis. Despite dental mobility, no extractions were required. The procedure was completed without complications, and postoperative healing was satisfactory. The smear obtained from the FNA revealed a representative sample showing large, round cells with frequent mitotic figures suggestive of lymphoma. Histopathological examination of the biopsy specimen revealed fibrous connective tissue densely infiltrated by mononuclear lymphocytes. Immunohistochemical analysis subsequently showed positivity for LCA, CD20, PAX5, CD10, and BCL‐6 in more than 30% of the cells, and BCL‐2 in fewer than 30% of the cells. A high proliferative index was identified (Ki‐67: 80%). CD3 and MUM‐1 antibodies were negative (Figure 2). Based on these findings, a diagnosis of Diffuse Large B‐Cell Non‐Hodgkin Lymphoma was established. The immunohistochemical panel is summarized in Table 1.

**TABLE 1:** Immunohistochemical panel.

Antibody	Result
LCA	Positive
CD20	Positive
CD3	Negative
CD10	Positive
BCl‐6	Positive (>30%)
BCl‐2	Positive (<30%)
MUM‐1	Negative
PAX‐5	Positive
Antígeno Ki67	Alto (80%)

**FIGURE 3:**
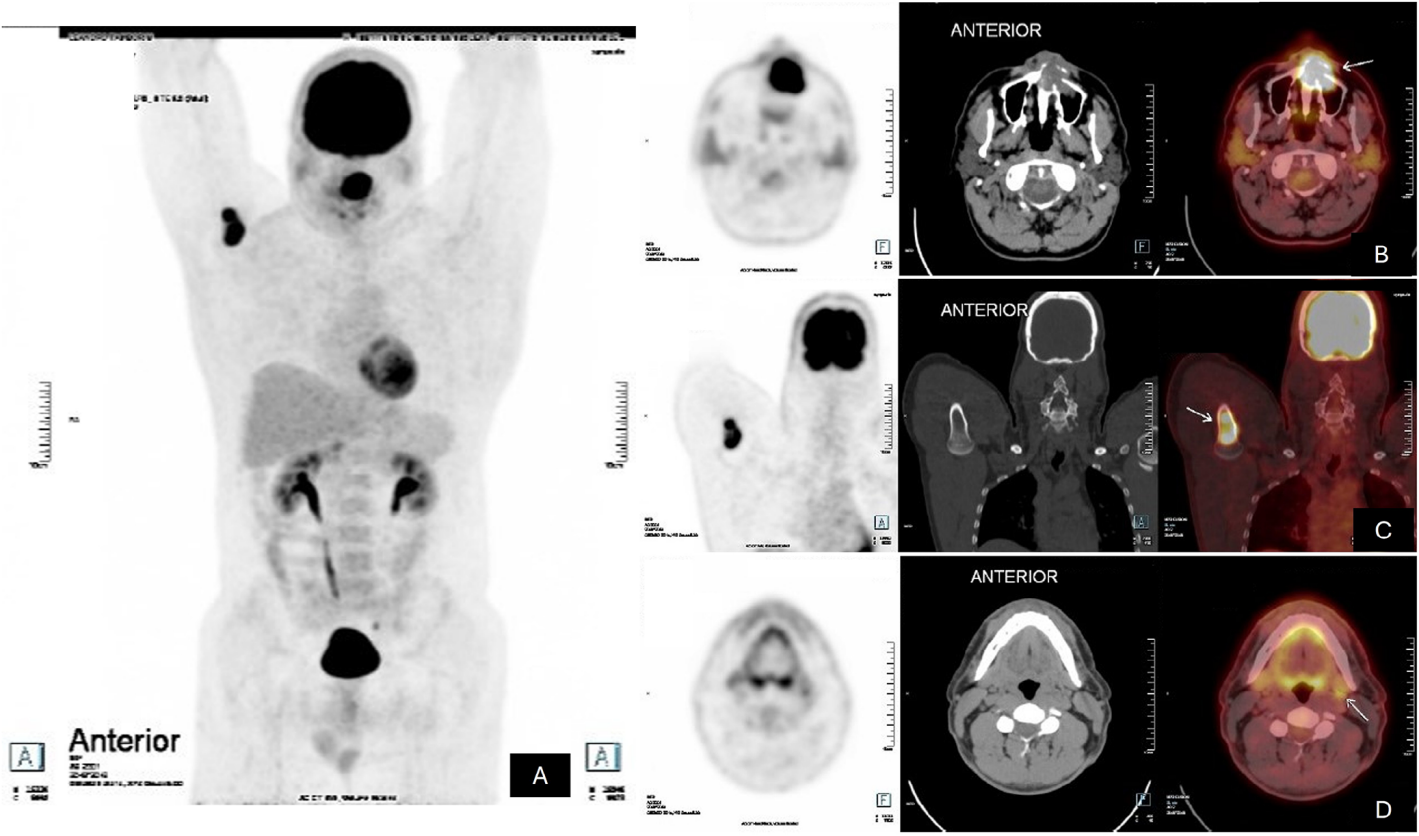
PET scan findings. (A) Whole‐body 18F‐FDG PET/CT scan revealing increased radiotracer uptake in the maxilla, right humerus, and left cervical lymph node. (B) PET/CT scan demonstrating a hypermetabolic lesion in the left maxilla, associated with bone destruction and invasion of the inferior portion of the nasal cavity and the ipsilateral maxillary sinus. (C) Hypermetabolic medullary lesion in the proximal third of the right humerus. (D) Hypermetabolic lesion in the left cervical lymph node.

Following the diagnosis, the patient was referred to an oncology service. During disease staging, a positron emission tomography‐computed tomography (PET/CT) scan demonstrated a hypermetabolic lesion in the left maxilla, with bone destruction and invasion into the inferior portion of the nasal cavity and ipsilateral maxillary sinus. In addition, other two lesions were identified in the proximal third of the right humerus and in the left cervical lymph node (Figure 3). The disease was classified as Ann Arbor stage IV A, with R‐IPI (Revised‐International Prognostic Index): 2 GOOD 80% 4Y PFS and CNS‐IPI (Central Nervous System‐International Prognostic Index): intermediate. The patient underwent six cycles of chemotherapy using the R‐CHOP regimen (Rituximab, Cyclophosphamide, Hydroxydaunorubicin, Vincristine sulfate, and Prednisone).

**FIGURE 4:**
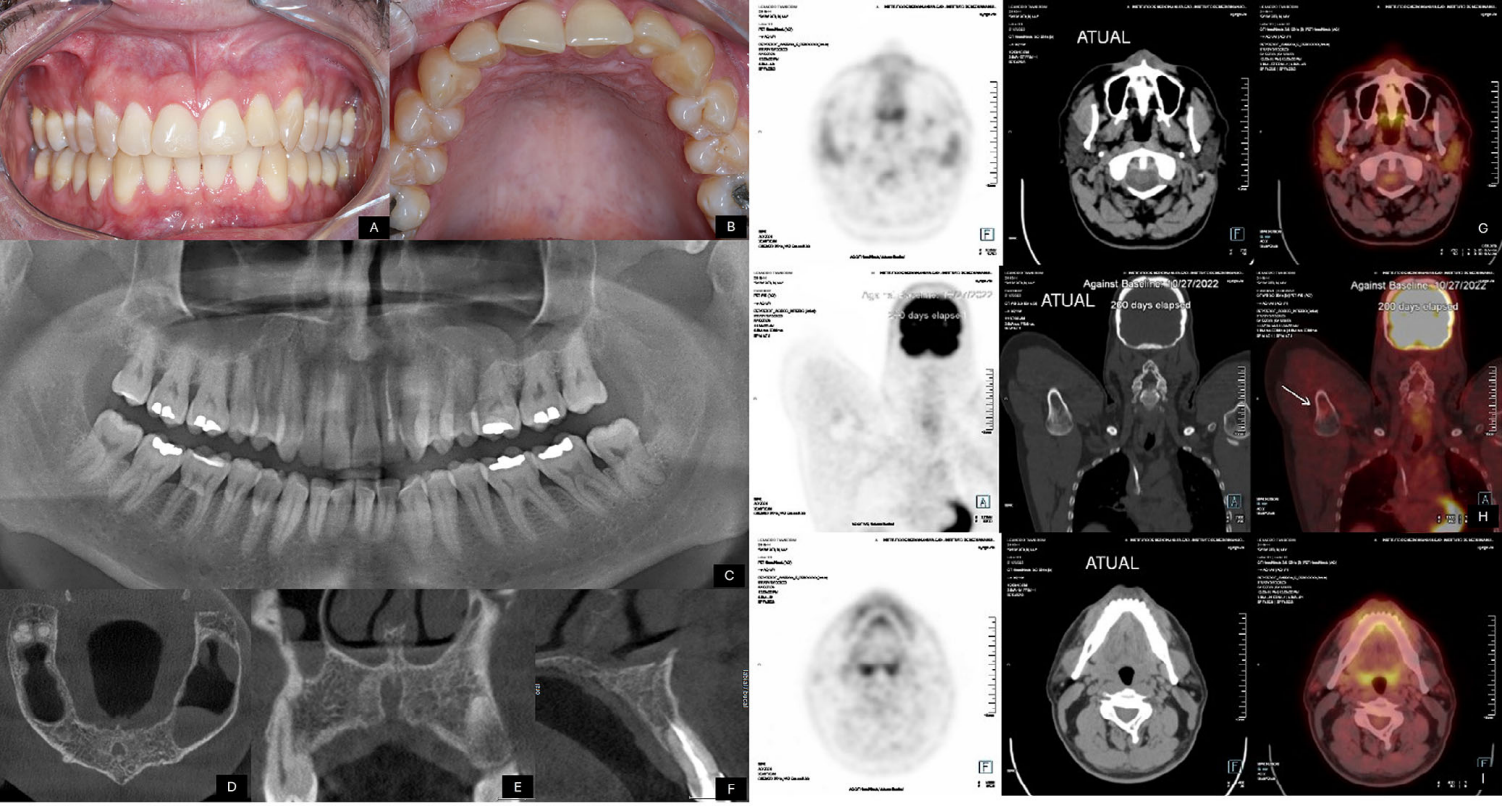
Clinical and radiographic assessment 18 months post‐treatment. (A‐B) Intraoral examination revealed intact, normochromatic oral mucosa, with no signs of swelling. The gingival “black space” in the interproximal region between central and lateral incisors had been successfully restored. (C) Panoramic radiograph demonstrating complete bone neoformation in the maxilla. (D‐F) Cone‐beam computed tomography (CBCT) scan confirming new bone formation in the anterior region of the left maxilla. (G‐I) PET/CT scan showing complete disease remission, with no evidence of hypermetabolic lesions in the maxilla, humerus, or cervical lymph node.

Eighteen months after completing the treatment, intraoral physical examination revealed intact and normochromatic oral mucosa, with no clinical signs of disease. Only a scar was observed in the maxillary vestibular sulcus, consistent with the previous biopsy, and the gingival black space in the interproximal region between central and lateral incisors had been restored. No dental mobility was observed. A panoramic radiograph and cone‐beam computed tomography (CBCT) scan showed new bone formation in the anterior region of the left maxilla. The PET/CT scan demonstrated complete remission of the lesions (Figure 4). The patient continues to undergo regular clinical and imaging surveillance, monitored by the Oral Medicine and Oncology teams.

The literature review identified 75 cases of lymphomas involving the periapical region (Table 2). Patient age ranged from 3 to 84 years, with a mean of 43,13 years. Regarding gender distribution, 32 patients were female, and 42 were male. The mandible was the most frequently affected site, accounting for 43 cases, while two patients presented simultaneous lesions in both the maxilla and mandible. The most prevalent subtype was diffuse large B‐cell lymphoma (DLBCL), representing 37 cases. Endodontic treatment was performed in 30 cases, whereas 16 underwent tooth extraction prior to the definitive lymphoma diagnosis.

**TABLE 2:** Literature review of cases affecting the periapical area.

Author	Age	Gender	Location	Diagnosis	Endodontic treatment	Time to diagnosis
Eisenbud et al., [8]	49	Female	Mandible	DLBCL	Yes	Several months
Eisenbud et al., 1984 [8]	44	Male	Mandible	DLBCL	Yes	NA
Eisenbud et al., 1984 [8]	29	Male	Mandible	Lymphocytic/small cell cleaved lymphoma	No^a^	2 months
Eisenbud et al., 1984 [8]	44	Female	Maxilla	DLBCL	No	NA
Eisenbud et al., 1984 [8]	42	Male	Maxilla	Histiocytic/Immunoblastic lymphoma	Yes	NA
Keyes et al., 1988 [9]	24	Male	Maxilla	Histiocytic lymphoma	Yes	3 months
Keyes et al., 1988 [9]	NA	Male	Mandible	Lymphoma	Yes	2 years
Wen et al., 1988 [10]	37	Male	Mandible	DLBCL	No	2 months
Spatafore et al., 1989 [11]	46	Male	Mandible	Lymphoma	No	1 year
Wannfors and Hammarström, 1990 [12]	45	Female	Mandible	Histiocytic lymphoma	Yes	NA
Gusenbauer et al., 1990 [13]	53	Male	Mandible	DLBCL	No^a^	Several months
Wright and Radman1, 1995 [14]	27	Male	Mandible	DLBCL	Yes	3 months
Mopsik and Milosbky, 1995 [15]	54	Male	Maxilla	Diffuse lymphoblastic lymphoma	Yes	1 year
Mopsik and Milosbky, 1995 [15]	59	Male	Mandible	DLBCL	Yes	4 months
Ardekian et al., 1999 [16]	16	Male	Mandible	Burkitt lymphoma	Yes	2 weeks
Kawasaki et al., 1997 [17]	60	Female	Mandible	NHL T	Yes	3 weeks
Liu et al., 2000 [18]	14	Male	Maxilla	Burkitt Lymphoma	No	3 weeks
Cheng and Wright, 2003 [19]	65	Female	Mandible	DLBCL	No	NA
Kozakiewicz et al., 2003 [20]	54	NA	Maxilla	B‐cell lymphoma	No^a^	NA
Brooks et al., 2005 [21]	29	Female	Maxilla	High grade B‐cell lymphoma	No	1 week
Kini et al., 2009 [22]	55	Male	Mandible	B‐cell lymphoma	No^a^	6 months
Balasubramaniam et al., 2009 [23]	36	Female	Mandible	Burkitt Lymphoma	No	4 weeks
Yamada et al., 2010 [24]	44	Male	Maxilla	Adult T‐cell leukemia/lymphoma	No	1 month
Saund et al., 2010 [25]	38	Female	Maxilla	Lymphoma	Yes	9 months
Agrawal et al., 2011 [26]	30	Female	Mandible	DLBCL	No^a^	2 weeks
Hopp et al., 2012 [27]	39	Male	Mandible	DLBCL	Yes	2 years
Fischer et al., 2012 [28]	34	Male	Maxilla	DLBCL	Yes	3 weeks
Jessri et al., 2013 [29]	32	Male	Mandible	DLBCL	Yes	3 months
Wong et al., 2013 [30]	50	Male	Maxilla	DLBCL	Yes	6 months
Wong et al., 2013 [30]	31	Female	Maxilla	DLBCL	Yes	2 months
Mendonca et al., 2013 [31]	38	Female	Mandible	DLBCL	Yes	Several months
Carbone et al., 2014 [32]	71	Female	Mandible	DLBCL	No^a^	NA
Pereira et al., 2015 [33]	48	Male	Mandible	DLBCL	Yes	5 months
Alshahrani et al., 2015 [34]	18	Male	Mandible	DLBCL	No	6 months
Bugshan et al., 2015 [35]	54	Male	Mandible	High grade B‐cell lymphoma	Yes	NA
Buchanan et al., 2015 [36]	35	Male	Maxilla	DLBCL	No	2 months
Tetik et al., 2016 [37]	43	Male	Mandible	DLBCL	No	1 month
Kumar et al., 2016 [38]	41	Female	Maxilla	DLBCL	Yes	4 months
Syed et al., 2016 [39]	81	Male	Maxilla	DLBCL	No^a^	1 month
Vasudevan et al., 2016 [40]	19	Female	Mandible	Plasmablastic lymphoma	No	3 months
Srikant et al., 2016 [41]	64	Male	Mandible	Spindle‐cell variant of NHL	No	6 months
MacDonald et al., 2017 [42]	59	Female	Maxilla	B‐cell lymphoma	Yes	NA
Dolan et al., 2017 [7]	68	Male	Maxilla/ Mandible	Marginal zone lymphoma / Lymphoplasmacytic lymphoma	No/Yes	4 months
Hassona et al., 2017 [43]	75	Male	Mandible	DLBCL	Yes	2 weeks
Varun et al., 2017 [44]	65	Male	Mandible	B‐cell lymphoma	No^a^	NA
Fuessinger et al., 2018 [45]	40	Female	Mandible	High grade B‐cell lymphoma	No	1 month
Zou et al., 2018 [46]	67	Female	Maxilla	DLBCL	No^a^	6 months
Cabras et al., 2018 [47]	15	Female	Maxilla	Burkitt Lymphoma	Yes	NA
Silva et al., 2018 [48]	29	Female	Mandible	THRLBCL	No	2 months
Lapthanasupkul et al., 2019 [49]	55	Female	Maxilla	ALCL	No	1 month
Janardhanan et al., 2019 [50]	32	Female	Maxilla	DLBCL	No^a^	NA
Siqueira et al., 2019 [51]	51	Male	Mandible	DLBCL	No^a^	4 months
Badabaan and Fatahzadeh, 2020 [52]	54	Male	Mandible	DLBCL	Yes	Few months
Karadwal et al., 2020 [53]	50	Male	Mandible	Diffused B‐cell lymphoma, mixed small and large cell type	No^a^	1 month
Theofilou et al., 2020 [54]	70	Female	Maxilla	DLBCL	No	3 years
Goutzanis et al., 2020 [55]	20	Male	Mandible	T‐LBL	No^a^	2 weeks
Shilkofski et al., 2020 [56]	72	Male	Maxilla	DLBCL	Yes	Few weeks
Silveira et al., 2020 [57]	14	Female	Maxilla	B‐cell Lymphoblastic lymphoma	No	1 month
Coskunses et al., 2020 [58]	82	Female	Mandible	DLBCL	No^a^	2 months
Nosrat et al., 2020 [59]	40	Female	Maxilla	DLBCL	Yes^a^	3 months
De Coninck et al., 2020 [60]	7	Female	Mandible	Burkitt Lymphoma	No	Few months
Parker, William D, and Keith [61]	37	Female	Mandible	Burkitt Lymphoma	No^a^	NA
Riaz et al., 2021 [62]	3	Male	Mandible	Burkitt Lymphoma	No	20 days
Vardas et al., 2022 [63]	84	Male	Maxilla	DLBCL	No^a^	15 days
Gante et al. 2022 [64]	44	Male	Mandible	DLBCL	No	4 months
Chait et al., 2023 [65]	7	Male	Maxilla	Burkitt Lymphoma	No	NA
Vyas et al., 2024 [66]	60	Male	Maxilla	DLBCL	No^a^	2 months
Shil et al., 2024 [67]	10	Female	Maxilla	T‐LBL	No	1 month
Polard et al., 2024 [68]	47	Male	Maxilla	High grade B‐cell lymphoma	No	2 years
Rezazadeh et al., 2024 [69]	18	Female	Maxilla	DLBCL	No	15 weeks
Gatt et al., 2024 [70]	32	Male	Maxilla	HTLV1+ T‐cell lymphoma	No	1 month
El Gaouzi et al., 2024 [71]	26	Female	Maxilla/ mandible	Burkitt Lymphoma	No	1 month
Papadopoulou et al., 2024 [72]	11	Female	Mandible	Burkitt Lymphoma	No	3 weeks
Wongpattaraworakul et al., 2025 [73]	61	Male	Maxilla	DLBCL	Yes	NA
Wongpattaraworakul et al., 2025 [73]	68	Female	Mandible	DLBCL	Yes	4 months

Abbreviations: ALCL, anaplastic large cell lymphoma; DLBCL, diffuse large B‐cell lymphoma; HTLV1+ T‐cell lymphoma, human T‐lymphotropic virus‐1 associated with adult T‐cell lymphoma; NA, not available; NHL T, non‐Hodgkin lymphoma T‐cell; THRLBCL, T‐cell/histiocyte‐rich large B‐cell lymphoma; T‐LBL, T‐cell lymphoblastic lymphoma.

^a^ Tooth extraction.

## 3 Discussion

This case report describes a rare presentation of diffuse large B‐cell lymphoma (DLBCL) initially manifesting as a persistent periapical lesion in the anterior maxilla, mimicking an endodontic pathology. The diagnostic complexity in distinguishing between endodontic and non‐endodontic periapical lesions is emphasized. Although the majority periapical radiolucencies are of endodontic origin and respond favorably to conventional root canal treatment, persistent cases should raise suspicion of an underlying malignancy [2].

Malignant non‐endodontic periapical lesions are rare diagnoses [3]. A literature review identified 30 distinct non‐endodontic pathological entities that mimicked inflammatory periapical lesions, with malignancies, including primary and metastatic tumors, accounting for 45.1% of cases [74]. However, a retrospective analysis of periapical biopsy specimens reported a lower incidence, with only 3.26% of cases classified as malignant [6].

Lymphomas account for approximately 5% of malignant neoplasms in the head and neck region, with half of these affecting the oral and maxillofacial structures [5]. Lymphomas are classified into two main categories based on histopathological characteristics, with non‐Hodgkin lymphoma (NHL) accounting for approximately 90% of cases, while Hodgkin lymphoma (HL) represents the remaining 10% [75, 76]. The different lymphoma subtypes are identified according to their cellular origin, including B‐cell lymphomas (BCL), T‐cell lymphoid tumors (TCL), natural killer/T‐cell lymphomas (T/NK‐NHL), and HL [77].

In our review, diffuse large B‐cell lymphoma (DLBCL) accounted for approximately 50% of the reported cases. This is the most prevalent subtype, including among periapical lymphomas [78, 79]. DLBCL, not otherwise specified (NOS), is an aggressive neoplasm characterized by large B‐cells arranged in a diffuse growth pattern [80]. Despite its aggressive behavior, DLBCL NOS is potentially curable and associated with a high survival rate (SR), particularly with early diagnosis and appropriate treatment [81]. A retrospective analysis of patients with DLBCL in the head and neck region revealed a 3‐year survival rate of 59.26%, with poor prognosis and increased risk of disease‐related mortality were observed exclusively in patients over 80 years old [75].

DLBCL, NOS involves extranodal sites in approximately 40% of cases, with the gastrointestinal tract being the most frequently affected, followed by bones, testes, spleen, thyroid, liver, kidneys, and adrenal glands [80]. In the head and neck, the most commonly involved site is the Waldeyer's ring, followed by the paranasal sinuses and oral cavity [76, 80]. Within the oral cavity and jawbones, the gingiva is the primary site of occurrence, though the palate is also a frequent location for early‐stage oral DLBCL, NOS [81]. Findings from our review demonstrated a higher frequency of mandibular involvement compared to the maxilla, with the mandible affected in over 57% of the cases analyzed.

When DLBCL NOS involves the jawbones, its clinical presentation is often nonspecific, mimicking endodontic, periodontal, and other osteolytic lesions [3, 5]. A systematic review evaluating leukemia and lymphoma cases that mimicked periapical lesions and led to endodontic treatment revealed that pain was present in 16.8% of cases, while edema was the most common clinical manifestation, affecting 35.8% of cases, followed by dental mobility (30.5%) [4]. The present case exhibited all these features, further complicating the initial diagnosis.

Although periapical lesions of neoplastic origin may share clinical features with endodontic pathologies, specific characteristics favor a malignant etiology. These include minimal caries, dental mobility without periodontal disease, regional paresthesia, cervical lymphadenopathy, and a lack of response to appropriate endodontic therapy [5, 27]. Neurological symptoms should prompt clinicians to investigate malignancy, as sensory disturbances, including numbness of the lips, tongue, cheek, and infraorbital region, often result from structural compromise of the mandibular canal, infraorbital nerve canal, or pterygopalatine fossa [82].

Radiographic assessment is fundamental in the diagnostic process. High‐quality radiographs and computed tomography (CT) scans are essential for accurately evaluating lesions and identifying subtle features that aid in differential diagnosis [79]. Lymphomas affecting the jaws are frequently associated with teeth, leading to root resorption and displacement. Additional radiographic findings include jaw expansion, locally destructive osteolytic lesions with poorly defined margins, and frequent cortical disruption [5, 79]. Ill‐defined lesion margins, uncommon in dental infections, may serve as radiographic indicators of malignancy [4, 78]. Additional radiographic patterns include a “moth‐eaten” appearance, lamellar periosteal bone formation, widening of the mandibular canal, irregular periodontal ligament space enlargement, and alterations in tooth spacing [5].

In cases of maxillary lymphoma, tumor progression may extend into adjacent anatomical structures, such as the maxillary sinus, hard palate, orbit, nasal cavity, pterygopalatine fossa, and infraorbital nerve canal [82]. In the present case, imaging demonstrated significant maxillary expansion with invasion into the maxillary sinus and nasal cavity, a pattern characteristic of aggressive neoplastic behavior. The presence of extensive bone destruction in these regions further reinforced the malignant nature of the lesion.

Similar to the present case, the literature indicates that periapical lymphomas are frequently misdiagnosed, as malignancy indicators are often overlooked, likely due to limited awareness that malignant neoplasms can mimic periapical conditions [4]. Studies show that nearly 49% of periapical lymphomas are initially mistaken for inflammatory periapical pathologies, leading to inappropriate endodontic treatment [5]. This finding supports the results of the review, which identified that 40% of misdiagnosed periapical lymphomas underwent endodontic treatment. This diagnostic delay can negatively impact prognosis and increase mortality rates [3, 4]. According to our findings, the time to definitive diagnosis ranged from 15 days to 2 years. In the current case achieved definitive diagnosis within approximately 3 months, emphasizing the importance of early suspicion.

This case underscores the necessity of considering malignancies in the differential diagnosis of persistent periapical lesions [4, 27]. Accurate clinic radiographic correlation, combined with histopathological evaluation, is essential for timely and precise diagnosis [79]. A thorough understanding of periapical malignancies and close collaboration among endodontists, pathologists, and oral medicine specialists are crucial for early diagnosis and improved patient outcomes.

## Author Contributions

**L.T.M.Q.C**.: Conceptualization, Writing – original draft, Writing – review & editing; **J.C.R**.: Conceptualization, Investigation; **I.S.P.F**.: Investigation; **S.M.P.L**.: Investigation; **A.R.S.‐S**.: Investigation; **P.A.V**.: Investigation; **M.A.L**.: Conceptualization, Project administration, Writing – review & editing.

## Funding

This work was supported in part by the Higher Education Personnel Improvement Coordination (CAPES), Ministry of Education, Brazil (Finance code 001—Grant number 88887.962729/2024‐00).

## Conflicts of Interest

The authors declare that they have no known competing financial interests or personal relationships that could have appeared to influence the work reported in this article.

## Ethics Statement

The study was conducted in accordance with the ethical principles established by the Institutional Research Ethics Committee, ensuring compliance with current ethical and scientific guidelines.

## Clinical Study Registration

This study is a case report with a literature review and does not involve a clinical trial or prospective clinical study. Therefore, a Clinical Study Registration Number is not applicable.

## Supporting information




**Supporting Information**: scd70162‐sup‐0001‐figure.png

## References

[1] T. Kosanwat , S. Poomsawat , and J. Kitisubkanchana , “Non‐Endodontic Periapical Lesions Clinically Diagnosed as Endodontic Periapical Lesions: A Retrospective Study Over 15 Years,” Journal of Clinical and Experimental Dentistry 13, no. 6 (2021): e586–e593, 10.4317/jced.57957. 34188765 PMC8223148

[2] K. Karamifar , A. Tondari , and M. A. Saghiri , “Endodontic Periapical Lesion: An Overview on Etiology, Diagnosis and Current Treatment Modalities,” European Endodontic Journal 5, no. 2 (2020): 54–67, 10.14744/eej.2020.42714. 32766513 PMC7398993

[3] L. F. Schuch and C. C. Vieira , “Uchoa Vasconcelos AC. Malignant Lesions Mimicking Endodontic Pathoses Lesion: A Systematic Review,” Journal of Endodontics 47 (2021): 178–188, 10.1016/j.joen.2020.08.023. 32918962

[4] M. de Queiroga IS , V. Z. Drumond , L. G. Abreu , et al., “Leukemia and Lymphoma Mimicking Periapical Conditions Resulting in Endodontic Treatment: A Systematic Review,” Journal of Endodontics 51 (2025): 106–117, 10.1016/j.joen.2024.11.005. 39577766

[5] H. Mortazavi , M. Baharvan , and K. Rezaeifar , “Periapical Lymphoma: Review of Reported Cases in the Literature,” Journal of Stomatology Oral and Maxillofacial Surgery 121 (2020): 404–407, 10.1016/j.jormas.2020.01.006. 32035143

[6] C. C. Vieira , F. G. Pappen , L. B. Kirschnick , et al., “A Retrospective Brazilian Multicenter Study of Biopsies at the Periapical Area: Identification of Cases of Nonendodontic Periapical Lesions,” Journal of Endodontics 46 (2020): 490–495, 10.1016/j.joen.2020.01.003. 32061420

[7] J. M. Dolan , A. DeGraft‐Johnson , N. McDonald , B. B. Ward , T. J. Phillips , and S. M. Munz , “Maxillary and Mandibular Non‐Hodgkin Lymphoma With Concurrent Periapical Endodontic Disease: Diagnosis and Management,” Journal of Endodontics 43 (2017): 1744–1749, 10.1016/j.joen.2017.04.001. 28734649

[8] L. Eisenbud , J. Sciubba , R. Mir , and S. A. Sachs , “Oral Presentations in Non‐Hodgkin's Lymphoma: A Review of Thirty‐One Cases. Oral Surgery,” Oral Medicine and Oral Pathology 57 (1984): 272–280, 10.1016/0030-4220(84)90183-X. 6584818

[9] G. G. Keyes , F. S. Balaban , and D. A. Lattanzi , “Periradicular Lymphoma: Differentiation From Inflammation. Oral Surgery,” Oral Medicine and Oral Pathology 66 (1988): 230–235, 10.1016/0030-4220(88)90099-0. 3174058

[10] B. Wen , M. K. Zahra , D. H. Hussey , J. Fred Doornbos , and A. Vigliotti , “Primary Malignant Lymphoma of the Mandible,” Journal of Surgical Oncology 39 (1988): 39–42, 10.1002/jso.2930390109. 3047499

[11] C. M. Spatafore , G. Keyes , and A. E. Skidmore , “Lymphoma: An Unusual Oral Presentation,” Journal of Endodontics 15 (1989): 438–441, 10.1016/S0099-2399(89)80179-7. 2637338

[12] K. Wannfors and L. Hammarström , “Periapical Lesions of Mandibular Bone: Difficulties in Early Diagnostics. Oral Surgery,” Oral Medicine and Oral Pathology 70 (1990): 483–489, 10.1016/0030-4220(90)90216-F. 2216386

[13] A. W. Gusenbauer , N. F. Katsikeris , and A. Brown , “Primary Lymphoma of the Mandible: Report of a Case,” Journal of Oral and Maxillofacial Surgery 48 (1990): 409–415, 10.1016/0278-2391(90)90442-5. 2179495

[14] J. M. WRIGHT and R. WP , “Intrabony Lymphoma Simulating Periradicular Inflammatory Disease,” Journal of the American Dental Association 126 (1995): 101–105, 10.14219/jada.archive.1995.0002. 7822634

[15] E. R. Mopsik and S. A. Milobsky , “Malignant Lymphoma Presenting as Periapical Pathology: A Report of Two Cases,” MSDA Journal 38 (1995): 175–179. 9569869

[16] L. Ardekian , A. Rachmiel , D. Rosen , I. Abu‐El‐Naaj , M. Peled , and D. Laufer , “Burkitt's Lymphoma of the Oral Cavity in Israel,” Journal of Cranio‐Maxillofacial Surgery 27 (1999): 294–297, 10.1054/jcms.1999.0074. 10717831

[17] G. Kawasaki , M. Nakai , A. Mizuno , T. Nakamura , and H. Okabe , “Malignant Lymphoma of the Mandible,” Oral Surgery, Oral Medicine, Oral Pathology and Oral Radiology Endodontology 83 (1997): 345–349, 10.1016/S1079-2104(97)90241-9. 9084197

[18] R. Liu , H. Liu , J. Bu , and S. Dong , “Burkitt's Lymphoma Presenting With Jaw Lesions,” Journal of Periodontology 71 (2000): 646–649, 10.1902/jop.2000.71.4.646. 10807131

[19] Y.‐S. L. Cheng and J. Wright , “Periapical Lymphoma,” Texas Dental Journal 120 (2003): 497–500. 12861904

[20] M. Kozakiewicz , M. Karolewski , J. W. Kobos , and Z. Stołecka , “Malignant Lymphoma of the Jaw Bone,” Medical Science Monitor 9 (2003): CS110–CS1104. 14646980

[21] J. K. Brooks , M. J. Ribera , C. W. Rogers , and B. L. Jurist , “Unusual Radiographic Presentations. Report of Five Cases,” New York State Dental Journal 71 (2005): 48–50. 16514879

[22] R. Kini , A. Saha , and V. Naik , “Diffuse Large B‐Cell Lymphoma of Mandible: A Case Report,” Medicina Oral, Patología Oral y Cirugía Bucal 14 (2009): e421–e424. 19718002

[23] R. Balasubramaniam , A. Goradia , L. N. Turner , et al., “Burkitt Lymphoma of the Oral Cavity: An atypical Presentation,” Oral Surgery, Oral Medicine, Oral Pathology and Oral Radiology Endodontology 107 (2009): 240–245, 10.1016/j.tripleo.2008.09.008. 19138642

[24] T. Yamada , K. Mishima , A. Ota , et al., “A Case of ATLL (Adult T‐Cell leukemia/lymphoma) Mimicking Odontogenic Infection,” Oral Surgery, Oral Medicine, Oral Pathology and Oral Radiology Endodontology 109 (2010): e51–e55, 10.1016/j.tripleo.2010.02.021. 20451832

[25] D. Saund , S. Kotecha , J. Rout , and T. Dietrich , “Non‐Resolving Periapical Inflammation: A Malignant Deception,” International Endodontic Journal 43 (2010): 84–90, 10.1111/j.1365-2591.2009.01644.x. 20002805

[26] M. Agrawal , S. Agrawal , and D. Kambalimath , “Non‐Hodgkins Lymphoma of Maxilla: A Rare Entity,” National Journal of Maxillofacial Surgery 2 (2011): 210, 10.4103/0975-5950.94485. 22639517 PMC3343395

[27] R. N. Hopp , M. T. Marchi , M. G. Kellermann , V. H. T. Rizo , M. A. Lopes , and J. Jorge , “Lymphoma Mimicking a Dental Periapical Lesion,” Leukemia & Lymphoma 53 (2012): 1008–1010, 10.3109/10428194.2011.631161. 22023527

[28] D. J. Fischer , G. D. Klasser , and R. Kaufmann , “Intraoral Swelling and Periapical Radiolucency,” Journal of the American Dental Association 143 (2012): 985–988, 10.14219/jada.archive.2012.0324. 22942144

[29] M. Jessri , A. A. Majeed , M. Matias , and C. Farah , “A Case of Primary Diffuse Large B‐Cell Non‐Hodgkin's Lymphoma Misdiagnosed as Chronic Periapical Periodontitis,” Australian Dental Journal 58 (2013): 250–255, 10.1111/adj.12056. 23713648

[30] G. B. Wong , S. Spadafora , N. Barbon , and M. Caputo , “Primary Extranodal B‐Cell Non‐Hodgkin Lymphoma Mimicking an Endodontic Lesion: Report of 2 Cases,” Journal (Canadian Dental Association) 79 (2013): d93. 24059491

[31] E. F. Mendonça , T. O. Sousa , and C. Estrela , “Non‐Hodgkin Lymphoma in the Periapical Region of a Mandibular Canine,” Journal of Endodontics 39 (2013): 839–842, 10.1016/j.joen.2013.01.007. 23683289

[32] M. Carbone , F. Della Ferrera , L. Carbone , G. Gatti , and M. Carrozzo , “Numb Chin Syndrome as First Symptom of Diffuse Large B‐Cell Lymphoma,” Case Reports in Dentistry 2014 (2014): 1–5, 10.1155/2014/413162. PMC4279430 25580308

[33] D. L. Pereira , D. T. Fernandes , A. R. Santos‐Silva , P. A. Vargas , O. P. de Almeida , and M. A. Lopes , “Intraosseous Non‐Hodgkin Lymphoma Mimicking a Periapical Lesion,” Journal of Endodontics 41 (2015): 1738–1742, 10.1016/j.joen.2015.06.001. 26234541

[34] F. A. A. Alshahrani , A. S. Aljabab , I. H. Motabi , A. Alrashed , and S. Anil , “Primary Diffuse Large B‐Cell Lymphoma Involving the Mandible,” Journal of Contemporary Dental Practice 16 (2015): 840–844, 10.5005/JP-JOURNALS-10024-1767. 26581467

[35] A. Bugshan , J. Kassolis , and J. Basile , “Primary Diffuse Large B‐Cell Lymphoma of the Mandible: Case Report and Review of the Literature,” Case Reports Oncology 8 (2015): 451–455, 10.1159/000441469. PMC4649735 26600778

[36] A. Buchanan , S. Kalathingal , J. Capes , and Z. Kurago , “Unusual Presentation of Extranodal Diffuse Large B‐Cell Lymphoma in the Head and Neck: Description of a Case With Emphasis on Radiographic Features and Review of the Literature,” Dentomaxillofacial Radiology 44, no. 3 (2015): 20140288, 10.1259/dmfr.20140288. 25421808 PMC4614159

[37] A. Tetik , C. Peskersoy , B. O. Koyuncu , M. C. Solmaz , and G. Saydam , “Primary Extranodal Non‐Hodgkin's Lymphoma Mimicking a Painful Gingival Swelling: A Case Report,” OALib 03 (2016): 1–7, 10.4236/oalib.1102363.

[38] M. S. Kumar , A. Gannepalli , A. Chandragiri , and K. Amarnath , “Diffuse Large B‐Cell Lymphoma of Maxilla—A Case Report of Late Relapse,” Journal of Clinical Diagnostic Research 10 (2016): ZC94–ZC99, 10.7860/JCDR/2016/16139.7695. 27190967 PMC4866265

[39] A. Syed and S. R. Singer , “Non‐Hodgkin' s Lymphoma in the Oral Cavity,” (2017). 27263143

[40] V. Vasudevan , Y. R. Kumar , P. Chavva , and S. Naina , “Intraoral Plasmablastic Non‐Hodgkin's Lymphoma Associated With Human Immunodeficiency Virus,” Indian Journal of Dental Research 27 (2016): 334–338, 10.4103/0970-9290.186229. 27411665

[41] N. Srikant , S. Yinti , M. Baliga , and H. Kini , “A Rare Spindle‐Cell Variant of Non‐Hodgkin's Lymphoma of the Mandible,” Journal Oral Maxillofacial Pathology 20 (2016): 129–132, 10.4103/0973-029X.180970. PMC4860914 27194875

[42] D. MacDonald , T. Li , S. F. Leung , J. Curtin , A. Yeung , and M. A. Martin , “Extranodal Lymphoma Arising Within the Maxillary Alveolus: A Case Report,” Oral Surgary Oral Medicine Oral Pathology and Oral Radiology 124 (2017): e233–e238, 10.1016/j.oooo.2017.04.015. 28624341

[43] Y. Hassona , G. Almuhaisen , A. Almansour , and C. Scully , “Lymphoma Presenting as a Toothache: A Wolf in Sheep's Clothing,” BMJ Case Reports 2017 (2017): 3–6, 10.1136/bcr-2016-218686. PMC5278327 28119440

[44] B. R. Varun , N. O. Varghese , T. T. Sivakumar , and A. P. Joseph , “Extranodal Non‐Hodgkin's Lymphoma of the Oral Cavity: A Case Report,” Iranian Journal of Medical Science 42 (2017): 407–411. PMC5523050 28761209

[45] M. Fuessinger , P. Voss , M. Metzger , C. Zegpi , and W. Semper‐Hogg , “Numb Chin as Signal for Malignancy‐Primary Intraosseous Diffuse Large B‐Cell Lymphoma of the Mandible,” Annals of Maxillofacial Surgery 8 (2018): 143, 10.4103/ams.ams_163_17. 29963443 PMC6018272

[46] H. Zou , H. Yang , Y. Zou , L. Lei , and L. Song , “Primary Diffuse Large B‐Cell Lymphoma in the Maxilla,” Medicine (United States) 97, no. 20 (2018): e10707, 10.1097/MD.0000000000010707. PMC5976336 29768336

[47] M. Cabras , P. G. Arduino , L. Chiusa , R. Broccoletti , and M. Carbone , “Case Report: Sporadic Burkitt Lymphoma Misdiagnosed as Dental Abscess in a 15‐Year‐Old Girl,” F1000Research 7 (2018): 1–8, 10.12688/F1000RESEARCH.16390.1. PMC7029763 32117562

[48] R. N. F. Silva , E. F. Mendonça , A. C. Batista , C. G. de Alencar R , R. A. Mesquita , and N. L. Costa , “T‐Cell/Histiocyte‐Rich Large B‐Cell Lymphoma: Report of the First Case in the Mandible,” Head and Neck Pathology 13 (2019): 711–717, 10.1007/s12105-018-0948-9. 30019325 PMC6854205

[49] P. Lapthanasupkul , K. Songkampol , K. Boonsiriseth , and N. Kitkumthorn , “Anaplastic Large Cell Lymphoma of the Palate: A Case Report,” Journal of Stomatology and Oral Maxillofacial Surgery 120 (2019): 172–175, 10.1016/j.jormas.2018.09.003. 30291889

[50] M. Janardhanan , R. Suresh , V. Savithri , and R. Veeraraghavan , “Extranodal Diffuse Large B Cell Lymphoma of Maxillary Sinus Presenting as a Palatal Ulcer,” BMJ Case Reports 12 (2019): 10–13, 10.1136/bcr-2018-228605. PMC6441260 30739092

[51] J. M. Siqueira , P. M. Fernandes , Oliveira ACF , J. Vassallo , A. de Alves F , and G. C. Jaguar , “Primary Diffuse Large B‐Cell Lymphoma of the Mandible,” Autopsy Case Reports 9, no. 3 (2019): e2019109, 10.4322/acr.2019.109. 31528626 PMC6709649

[52] M. Fatahzadeh , “Primary Diffuse Large B‐Cell Lymphoma of Mandible Masquerading as a Toothache,” Quintessence Int 51, no. 1 (2020): 50–55, 10.3290/j.qi.a43666. 31792469

[53] A. Karadwal , S. Chatterjee , K. Pathak , and R. Sabharwal , “Diffused Mixed B‐Cell Non‐Hodgkin Lymphoma of Mandible,” Journal of Oral Maxillofacial Pathology 24 (2020): 77, 10.4103/jomfp.JOMFP_310_18. PMC7069130 32189910

[54] V. I. Theofilou , N. Katsoulas , K. I. Tosios , A. Sklavounou , and N. G. Nikitakis , “Richter Transformation in the Oral and Maxillofacial Area: Report of 2 Cases and Literature Review,” Oral Surgery Oral Medicine Oral Pathology and Oral Radiology 131 (2021): e14–e20, 10.1016/j.oooo.2020.02.016. 32402567

[55] L. Goutzanis , J. Apostolidis , C. Giatra , E. Chrysomali , and D. Deskos , “A Case of Systemic Precursor T‐cell Lymphoblastic Lymphoma Presenting With Single Tooth Mobility,” SAGE Open Medicine Case Reports 8 (2020): 2050313×2092796, 10.1177/2050313x20927961. PMC7273617 32547763

[56] J. A. Shilkofski , O. A. Khan , and N. K. Salib , “Non‐Hodgkin's Lymphoma of the Anterior Maxilla Mimicking a Chronic Apical Abscess,” Journal of Endodontics 46 (2020): 1330–1336, 10.1016/j.joen.2020.06.010. 32565334

[57] H. A. Silveira , L. M. Sousa , E. V. Silva , et al., “Primary Intraosseous CD9‐Positive B‐Cell Lymphoblastic Lymphoma of the Maxilla Affecting a Pediatric Patient: Immunohistochemical and In Situ Hybridization Analysis,” Oral Oncology 108 (2020): 9–12, 10.1016/j.oraloncology.2020.104910. 32771332

[58] F. M. Coskunses , Ü. Cilasun , P. Celik Topcu , and B. Tokuc , “Primary Diffuse Large B‐Cell Lymphoma of the Mandible: A Case Report,” Gerodontology 37 (2020): 307–311, 10.1111/ger.12470. 32809252

[59] A. Nosrat , P. Verma , S. Glass , C. E. Vigliante , and J. B. Price , “Non‐Hodgkin Lymphoma Mimicking Endodontic Lesion: A Case Report With 3‐Dimensional Analysis, Segmentation, and Printing,” Journal of Endodontics 47 (2021): 671–676, 10.1016/j.joen.2021.01.002. 33493549

[60] W. De Coninck , D. Govaerts , M. Bila , et al., “Burkitt Lymphoma in Children Causing an Osteolytic Lesion in the Mandible: A Case Report,” Clinical Case Reports 9 (2021): 938–943, 10.1002/ccr3.3703. 33598276 PMC7869388

[61] W. D. Parker and K. Jones , “Burkitt's Lymphoma: An Unexpected Cause of Dental Pain,” Journal of Surgery and Case Reports 2021 (2021): 1–4, 10.1093/jscr/rjaa557. PMC7884023 33613961

[62] N. Riaz , T. Saeed , and M. Nadeem , “Burkitt's Lymphoma of Mandible in a Young Pakistani Boy: A Case Report,” Jpma the Journal of the Pakistan Medical Association 71 (2021): 2265–2267, 10.47391/JPMA.04-655. 34580528

[63] E. Vardas , M. Georgaki , E. Papadopoulou , et al., “Diagnostic Challenges in a Diffuse Large B‐Cell Lymphoma of the Maxilla Presenting as Exposed Necrotic Bone,” Journal of Clinical and Experimental Dentistry 14 (2022): 303–309, 10.4317/jced.59346. PMC8916603 35317293

[64] J. Gante , S. Georg , J. M. G. Robez , A. Dubuc , and F. Lauwers , “Primary Extra‐Nodal Non‐Hodgkin's Lymphoma Affecting Mandibular Bone: A Case Report,” Pan African Medical Journal 41 (2022): 231, 10.11604/pamj.2022.41.231.33778. 35721648 PMC9167483

[65] F. Chait , N. Bahlouli , K. Laasri , et al., “A Dental Extraction Revealing a Multisystem Burkitt's Lymphoma: A Case Report,” Global Pediatric Health 11 (2024): 1–6, 10.1177/2333794x241227704. PMC10807309 38269317

[66] A. Vyas , A. Chug , A. Sehrawat , S. Simre , J. Porwal , and P. Kolse , “Maxillary Diffuse Large B‐Cell Lymphoma Enshrouded as Osteomyelitis: A Case Report,” Journal of Maxillofacial and Oral Surgery 23 (2024): 1022–1025, 10.1007/s12663-024-02174-9. 39118903 PMC11303643

[67] M. Shil , V. Srinivasan , A. A. Vincent , T. Gaikwad , and D. Thomas , “A Rare Case of T‐Cell Lymphoblastic Lymphoma: A Diagnostic Predicament,” Cureus 16, no. 8 (2024): e65994, 10.7759/cureus.65994. 39221387 PMC11366176

[68] P.‐L. Polard , A. Tempescul , and K. Vallaeys , “When Maxillofacial CBCT Permits Fortuitously to Diagnose Primary Non‐Hodgkin's Lymphoma: A Case Report,” Journal of Belgian Social Radiology 108 (2024): 1–6, 10.5334/jbsr.3682. PMC11378706 39246327

[69] F. Rezazadeh , Z. Mansouri , A. Sookhakian , and V. Mohammadkarimi , “Uncommon Presentation of Diffuse Large B‐Cell Lymphoma: Oral and Pulmonary Involvements in a Young Patient: A Case Report,” Journal of Medical Case Reports 18 (2024): 478, 10.1186/s13256-024-04825-4. 39407343 PMC11481727

[70] G. P. Le , P. M , P. Reimbold , I. Boussen , G. Lescaille , and J. Rochefort , “Human T‐Lymphotropic Virus‐1 Associated With Adult T‐Cell Lymphoma: A Case Report Case Presentation Case History and Examination,” Cureus 16 (2024): 1–7, 10.7759/cureus.73769. PMC11646550 39677084

[71] R. El Gaouzi , L. Benjelloun , and B. Taleb , “Atypical Presentation of Oral Burkitt Lymphoma in an Adult: A Case Report,” Journal of Medical Case Reports 18, no. 1 (2024): 618, 10.1186/s13256-024-04728-4. 39696685 PMC11658111

[72] E. Papadopoulou , M. Kouri , D. Velonis , et al., “Sporadic Burkitt Lymphoma First Presenting as Painful Gingival Swellings and Tooth Hypermobility: A Life‐Saving Referral,” Dental Journal 13 (2025): 1–20, 10.3390/dj13010006. PMC11764000 39851582

[73] W. Wongpattaraworakul , T. Krongbaramee , E. A. Lanzel , J. W. Hellstein , and F. B. Teixeira , “Hematolymphoid Neoplasm Mimicking Endodontic Lesions: Case Series and Review of the Literature,” Oral Surgery Oral Medicine Oral Pathology and Oral Radiology 139 (2024): e1–12, 10.1016/j.oooo.2024.07.011. 39179451

[74] S. Pimenta Carvalho , C. Estrela , and E. Franco Vencio , “Clinical Differential Diagnosis Between Nonodontogenic and Endodontic Radiolucent Lesions in Periapical Location: A Critical Review,” Iran Endodontic Journal 16 (2021): 150–157, 10.22037/iej.v16i3.32572. PMC9735257 36704403

[75] P. S. Bernardo , T. Hancio , F. Vasconcelos , et al., “Primary Diffuse Large B‐Cell Lymphoma of the Head and Neck in a Brazilian Single‐Center Study,” Oral Diseases 29 (2023): 968–977, 10.1111/odi.14104. 34905288

[76] S. Yan , J. Ma , M. Yang , et al., “Analysis of the Clinicopathologic Characteristics and Prognosis of Head and Neck Lymphoma,” Analytical Cellular Pathology 2022 (2022): 1–13, 10.1155/2022/4936099. PMC8888118 35242496

[77] K. Storck , M. Brandstetter , U. Keller , and A. Knopf , “Clinical Presentation and Characteristics of Lymphoma in the Head and Neck Region,” Head and Face Medicine 15, no. 1 (2019): 1, 10.1186/s13005-018-0186-0. 30606206 PMC6317257

[78] Y. Michi , H. Harada , Y. Oikawa , et al., “Clinical Manifestations of Diffuse Large B‐Cell Lymphoma That Exhibits Initial Symptoms in the Maxilla and Mandible: A Single‐Center Retrospective Study,” BMC Oral Health 22 (2022): 20, 10.1186/s12903-022-02056-x. 35081952 PMC8793180

[79] N. R. Gomes , L. A. Lima , A. L. Morais‐Perdigão , et al., “Radiological Aspects of Lymphomas and Leukaemias Affecting the Jaws: A Systematic Review,” Journal of Oral Pathology & Medicine 52 (2023): 315–323, 10.1111/jop.13422. 36852531

[80] C. I. Rodrigues‐Fernandes , A. G. Junior , C. D. Soares , et al., “Oral and Oropharyngeal Diffuse Large B‐Cell Lymphoma and High‐Grade B‐cell Lymphoma: A Clinicopathologic and Prognostic Study of 69 Cases,” Oral Surgery Oral Medicine Oral Pathology and Oral Radiology 131 (2021): 452–462.e4, 10.1016/j.oooo.2020.11.005. 33610538

[81] C. I. Rodrigues‐Fernandes , L. L. de Souza , and S.‐C. S. F. dos , “Clinicopathological Analysis of Oral Diffuse Large B‐Cell Lymphoma, NOS: A Systematic Review,” Journal of Oral Pathology & Medicine 48 (2019): 185–191, 10.1111/jop.12802. 30414287

[82] Y. Liu , Y. Wang , P. Wang , and Q. Yu , “A Retrospective Study to Evaluate the CT and MR Imaging Findings of Non‐Hodgkin's Lymphoma Affecting the Jaw Bones,” Oral Radiology 38 (2022): 509–516, 10.1007/s11282-021-00582-y. 35032248

