# Supplementary figures and images for: Intraosseous Non‐Hodgkin Lymphoma Mimicking a Periapical Lesion
Source: Spec Care Dentist. 2026 Mar 27;46(2):e70162. doi: 10.1111/scd.70162 (PMC13022747; doi:10.1111/scd.70162)

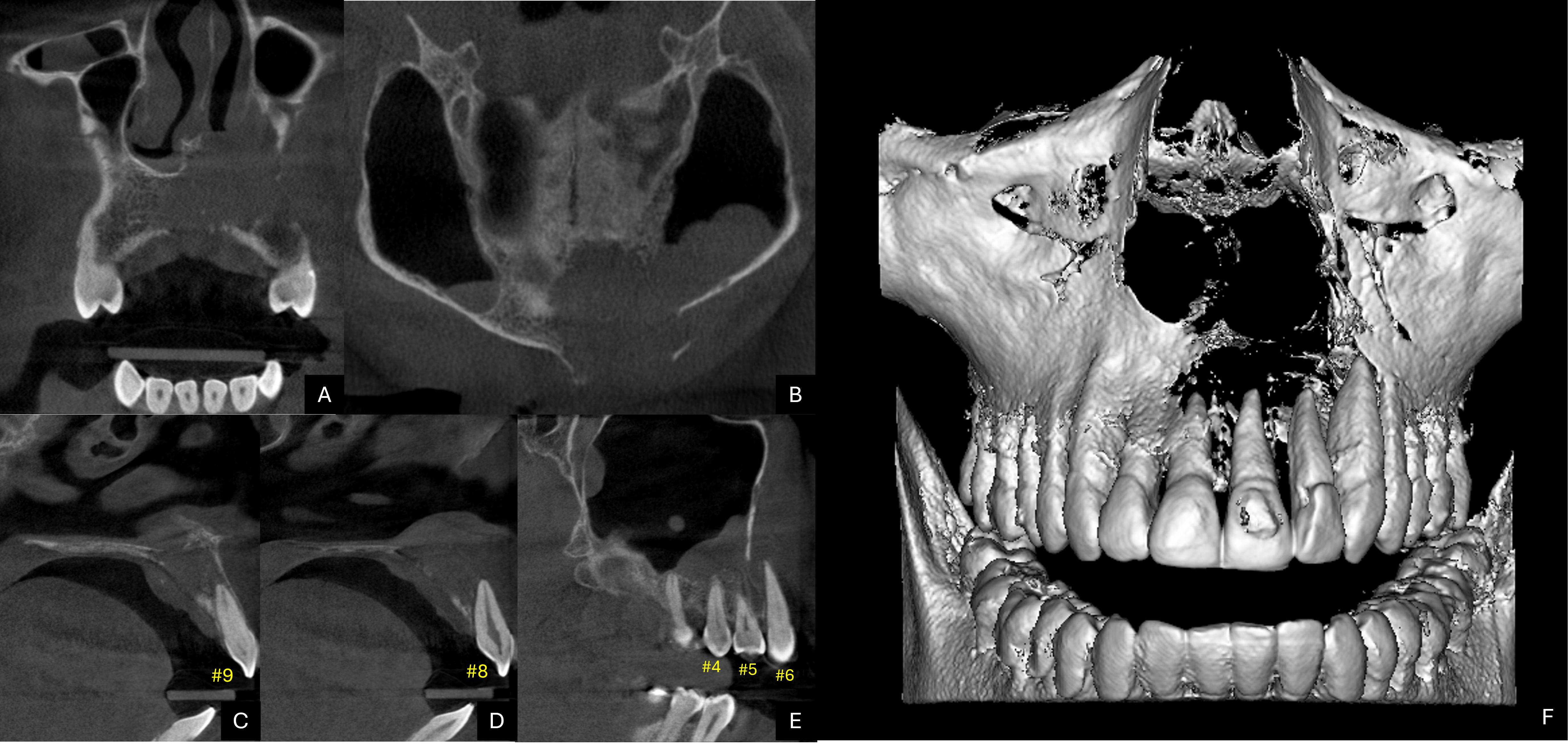

Supplement: Supplementary file 1 — Supporting Information: scd70162‐sup‐0001‐figure.png [file SCD-46-0-s001.png]
